# Transcriptome analysis reveals the mechanism of NaHCO_3_ promoting tobacco leaf maturation

**DOI:** 10.1515/biol-2022-0849

**Published:** 2024-04-15

**Authors:** Tingting Wang, Yuanyuan Zhao, Dexun Wang, Hongzhi Shi

**Affiliations:** College of Tobacco Science, Henan Agricultural University, No. 218 Pingan Avenue, Zhengdong New District, Zhengzhou, Henan, 450046, China; Dali Branch of Yunnan Provincial Tobacco Company, Dali, Yunnan, 671000, China

**Keywords:** tobacco, NaHCO_3_, RNA sequencing, leaf maturation

## Abstract

NaHCO_3_ accelerates the aging of tobacco leaves; however, the underlying molecular mechanisms have not been elucidated. This study aimed to explore the mechanism of NaHCO_3_ in the promotion of tobacco leaf maturation using transcriptome analysis. Leaves on plants or detached leaves of the tobacco variety, Honghua Dajinyuan, were sprayed with or without 1% NaHCO_3_. The leaf yellowing was observed, the pigment content and enzyme activities were determined and RNA sequencing (RNA-seq) was performed. Spraying NaHCO_3_ onto detached leaves was found to promote leaf yellowing. Pigment content, catalase activity, and superoxide dismutase activity significantly decreased, whereas peroxidase activity and malondialdehyde content significantly increased. RNA-seq demonstrated that spraying with NaHCO_3_ upregulated genes associated with cysteine and methionine metabolism; alpha-linolenic acid metabolism; and phenylalanine, tyrosine, and tryptophan biosynthesis and downregulated genes related to photosynthesis and carotenoid biosynthesis. Genes correlated with autophagy-other, valine, leucine, and isoleucine degradation, and the MAPK signaling pathway were upregulated while those correlated with DNA replication, phenylalanine, and tyrosine and tryptophan biosynthesis were downregulated in detached leaves sprayed with NaHCO_3_ compared with the plant leaves sprayed with NaHCO_3_. Overall, this study is the first to elucidate the molecular and metabolic mechanisms of NaHCO_3_ in the promotion of tobacco leaf maturation.

## 1 Introduction

Flue-cured tobacco is an important cash crop. Timely harvesting and moderate flue curing are key to ensuring the quality of this crop. Maturity is the primary quality factor for grading and a critical index for measuring the quality of tobacco leaves [1]. Insufficient maturity leads to weak hydrolytic activity of substances in the leaves, which causes difficulties during browning and flue curing. The hydrolysis of organic matter is intense in overripe leaves. As a result, the leaves turn yellow, dehydrate quickly, and are easy but not resistant to flue curing. Moderately mature tobacco leaves are easy to cure and have an ideal resistance to flue curing. In addition, the aroma content of these leaves increases with maturity, and the quality of flue-cured tobacco is good [2,3]. The upper leaves of tobacco have a high economic value; however, because the weather is not suitable for maturity later in the growing season, obtaining suitably mature and flue-cured high-quality tobacco is difficult to achieve [4]. Therefore, the degradation of substances in flue-cured tobacco must be promoted to enable rapid maturation of the leaves.

Currently, studies on the promotion of tobacco leaf ripening and yellowing have mainly focused on soil fertilizers and irrigation management [2]. Although this approach is a good strategy for accurately determining the amount of nitrogen fertilizer applied and the basic consumption of nitrogen fertilizer in the mature stage of tobacco leaves, controlling this system during the actual production process is not an easy task. Moreover, excessive nutrient consumption through delayed topping can promote the yellowing of tobacco leaves; however, delayed topping affects the quality of tobacco leaves [5]. Currently, only few reports have been published on the effects of chemical spraying on the ripening and yellowing of tobacco leaves. In nature, tobacco ripening is climacteric, characterized by high respiration and ethylene production [6]. Ethylene effectively induces leaf senescence and nicotine conversion [7]. A previous study revealed that treatment with 2-chloroethylphosphonic acid, which releases free ethylene, rapidly yields mature flue-cured tobacco leaves prior to harvest [8]. NaHCO_3_ affects the production of ethylene [5]. NaHCO_3_ stress also induces different degrees of chlorosis and yellowing [9,10] and accelerates the aging of tobacco leaves [5]. NaHCO_3_ is more convenient and cost-effective than ethylene. Moreover, NaHCO_3_ can induce nicotine demethylase activity in mature and senescent tobacco leaves after picking, promote the conversion of nicotine to nornicotine, and enable early identification of transformed strains [6]. Therefore, NaHCO_3_ may be a good exogenous substance to promote the maturation of tobacco leaves.

Owing to the major environmental challenges of soil salinity-alkalinity limiting crop productivity [11], some studies have reported global transcriptome changes following NaHCO_3_ treatment. For example, Ge et al. conducted transcriptional profiling of *Glycine soja* roots subjected to 50 mmol/L NaHCO_3_ treatment and found that most differentially expressed genes (DEGs) were involved in signal transduction, energy, transcription, secondary metabolism, transporters, disease, and defense responses [12]. Wang et al. found that the DEGs between *Tamarix hispida* roots treated and untreated with NaHCO_3_ were involved in signal transduction, phosphatase activity, and lipid kinase activity [13]. However, the molecular mechanism whereby NaHCO_3_ promotes the maturity of late-maturing tobacco leaves remains unclear.

In this study, we aimed to explore the mechanisms of NaHCO_3_ in the promotion of tobacco leaf maturation using transcriptome analysis. Accordingly, we treated tobacco leaves with NaHCO_3_ before and after picking and then conducted RNA sequencing (RNA-seq) and bioinformatics analysis.

## 2 Methods

### 2.1 Planting and sampling

The pot experiment was conducted at the Key Laboratory of Tobacco Cultivation, Physiology, and Biochemistry, Henan Agricultural University (Zhengzhou, Henan, China). The tobacco variety, Honghua Dajinyuan, was flue-cured in this study. After the tobacco seeds were disinfected, the seedlings were raised by floating. After four true leaves appeared, strong seedlings with uniform growth were selected and transplanted into pots filled with nutrient soil. The light, temperature, water, and fertilizer conditions were the same throughout the growth process of the tobacco plant. The temperature was set to 23–28°C, the light cycle was 10 h from 8:00 to 18:00 with a light intensity of 400 μmol m^−2^ s^−1^, and the humidity was 70 ± 5%. After the seedlings adapted to the pot environment, liquid fertilizer was applied to promote the growth of the tobacco plants.

At week 10, 24 pots with vigorous and consistent tobacco plant growth were selected and randomized into four treatments: the LT group was sprayed with 1% NaHCO_3_ solution after leaf picking (picking +1% NaHCO_3_); LT-CK was sprayed with the same volume of distilled water after leaf picking (picking + distilled water). The HT group was sprayed with 1% NaHCO_3_ solution on tobacco leaves that were not picked from tobacco plants (unpicking +1% NaHCO_3_); the CK group was sprayed with the same volume of distilled water, and the levels were not picked (unpicking + distilled water). Leaves from the LT and LT-CK groups were placed in a constant temperature (30°C) and humidity (80%) incubator to maintain leaf activity. Eight days after treatment, the leaves were sampled and immediately stored at −80°C for the subsequent experiments.

### 2.2 Physiological index detection

The chloroplast pigment content was determined using spectrophotometry. Leaf tissues were ground in liquid nitrogen. Acetone was added for pigment extraction overnight and then centrifuged at 10,000 × *g* for 10 min. The optical density values at 663, 646, and 470 nm were measured. The Chl a, Chl b, and carotenoid contents were determined according to a previous study [14].

Catalase (CAT), peroxidase (POD), and superoxide dismutase (SOD) activities were determined via ultraviolet absorption spectrometry using enzyme activity kits (Suzhou Comin Biotechnology Co., Ltd., Jiangsu, China). Malondialdehyde (MDA) content was detected using the glucosinolate barbituric acid colorimetric method.

### 2.3 RNA extraction and library construction

Total RNA was isolated from samples using TRIzol reagent (Thermo Fisher Scientific, Waltham, MA, USA). After quality confirmation, the RNA samples were subjected to cDNA library construction. In detail, mRNA with a poly(A) tail was enriched by magnetic beads with oligo(dT), and the mRNA was interrupted using a buffer solution. Using the fragmented mRNA as a template and random oligonucleotides as primers, the first cDNA strand was synthesized using the M-MuLV reverse transcriptase system. The second cDNA strand was synthesized using a DNA polymerase I system with dNTPs as the raw material. Purified double-stranded cDNA was subjected to end repair, A-tail addition, and joint connection sequencing. A cDNA of approximately 200 bp was screened for PCR amplification using AMPure XP beads. The PCR products were purified using AMPure XP beads. The library was obtained and subjected to Illumina sequencing following the quality control procedures.

### 2.4 Data processing

To ensure data quality, the raw reads were subjected to quality control using fastp (version 0.19.6) [15]. Briefly, reads containing adapter were removed, reads with more than 10% N were removed, reads that were all A-base were removed, and low-quality reads (base numbers with mass value of *Q* ≤ 20 accounting for more than 50% of the entire read) were removed. The clean reads were subjected to reference genome alignment to *Nicotiana tabacum* K326 genome, published by Sierro et al. [16], using HISAT2 (version 2.2.1) [17].

### 2.5 Sample relationship analysis and differential expression analysis

Based on the expression levels in each sample, principal component analysis (PCA) was conducted to determine the repeatability of the samples.

Differential gene expression analysis was conducted using DESeq2 (version 2.12) [18] based on read count data. The analysis was divided into the following three parts: (1) normalization of the read count, (2) *P*-value calculation, and (3) multiple hypothesis test correction to obtain the false discovery rate (FDR) value. The thresholds were FDR < 0.05 and |log_2_FC| > log_2_(2). Significant DEGs are displayed on a volcano map. The DEGs were also subjected to hierarchical clustering, and the clustering results are presented using heat maps.

### 2.6 Function analysis

Gene ontology (GO) and Kyoto Encyclopedia of Genes and Genomes (KEGG) enrichment analyses were performed to determine the functions of these DEGs. GO comprises three ontologies: molecular function (MF), cellular component (CC), and biological process (BP). Pathway enrichment analysis was conducted with the KEGG pathway as the unit. A hypergeometric test was used to select significantly enriched pathways.

### 2.7 Gene set enrichment analysis (GSEA)

Enrichment analysis based on traditional hypergeometric tests requires a set of significant DEGs. When individual gene changes are weak, the results based on traditional enrichment analysis may be limited. GSEA (Version 4.2.0) [19] can effectively compensate for the lack of effective information mining of minor genes using traditional enrichment analysis and can more comprehensively explain the regulatory effects of certain functional units, such as pathways or GO terms. Using the expression information of all genes, the genes were ranked according to Signal2Noise. After analysis of the ranking of a specific gene set among all genes, the GO term or pathway in which the gene set resides was scored; this score is called the enrichment score (ES). Based on the gene set, the *P*-value was calculated using a permutation test, and the normalized ES value (NES value) was corrected using multiple tests.

## 3 Results

### 3.1 NaHCO_3_ induces the senescence of detached tobacco leaves

**Figure 1:**
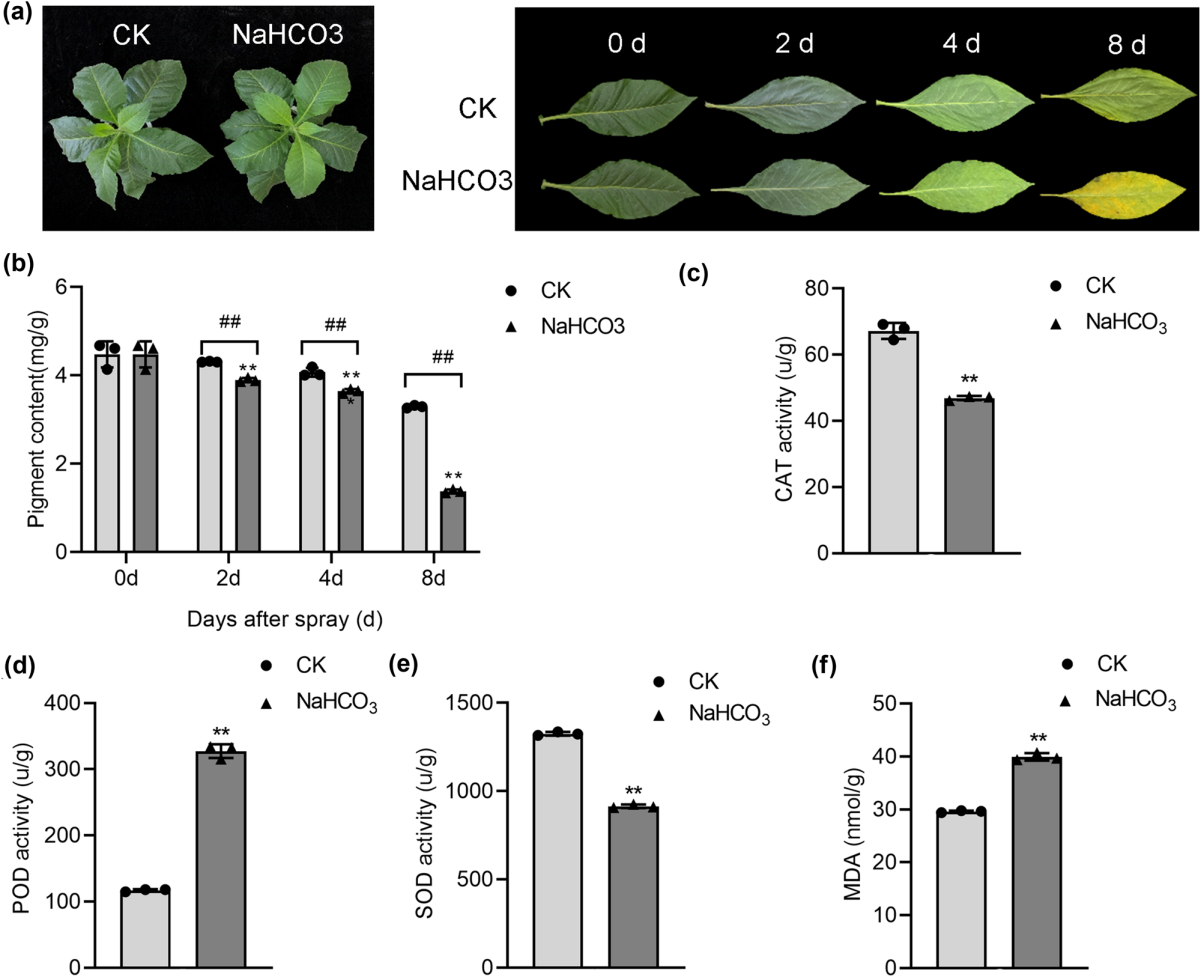
Changes in the physical characteristics of leaves after spraying with NaHCO_3_. (a) Appearance of live leaves on day 8 and detached leaves from days 0 to 8. (b) Effect of spraying NaHCO_3_ on the pigment content of detached flue-cured tobacco leaves. **P < 0.01, compared with the same treatment on day 0; ^##^ *P* < 0.01, compared with the CK group. (c) Effect of spraying NaHCO_3_ on the CAT activity of detached flue-cured tobacco leaves. **P < 0.01, compared with the CK group. (d) Effect of spraying NaHCO_3_ on the POD activity of detached flue-cured tobacco leaves. **P < 0.01, compared with the CK group. (e) Effect of spraying NaHCO_3_ on the SOD activity of detached flue-cured tobacco leaves. **P < 0.01, compared with the CK group. (f) Effect of spraying NaHCO_3_ on the MDA activity of detached flue-cured tobacco leaves. **P < 0.01, compared with the CK group.

The effect of spraying NaHCO_3_ on live tobacco leaves was not evident (Figure 1a) whereas that of spraying on detached leaves was evident. On day 2, the leaf tips and edges began to appear green. On day 4, the leaf surfaces began to transition from a green to a yellow color. More than 95% of the leaf surface became yellow on day 8, whereas only the leaf tip and edge began to turn yellow in the CK group (Figure 1a). The pigment content decreased in a time-dependent manner in the CK and NaHCO_3_ spraying groups. At the same time point, the pigment content of leaves in the NaHCO_3_ spraying group was significantly lower than that of the CK group (*P* < 0.01) (Figure 1b). The activities of CAT and SOD significantly decreased (*P* < 0.01), whereas those of POD and MDA significantly increased after NaHCO_3_ treatment (*P* < 0.01) (Figure 1c–f).

### 3.2 Data processing

For all samples, the raw sequencing data ranged from 40,061,984 to 48,891,692. Following removal of the low-quality data, clean data were obtained, accounting for more than 99% of all samples. After mapping to the reference genome, the total mapped genome ranged from 92.43 to 93.74%.

### 3.3 PCA and differential expression analysis

**Figure 2:**
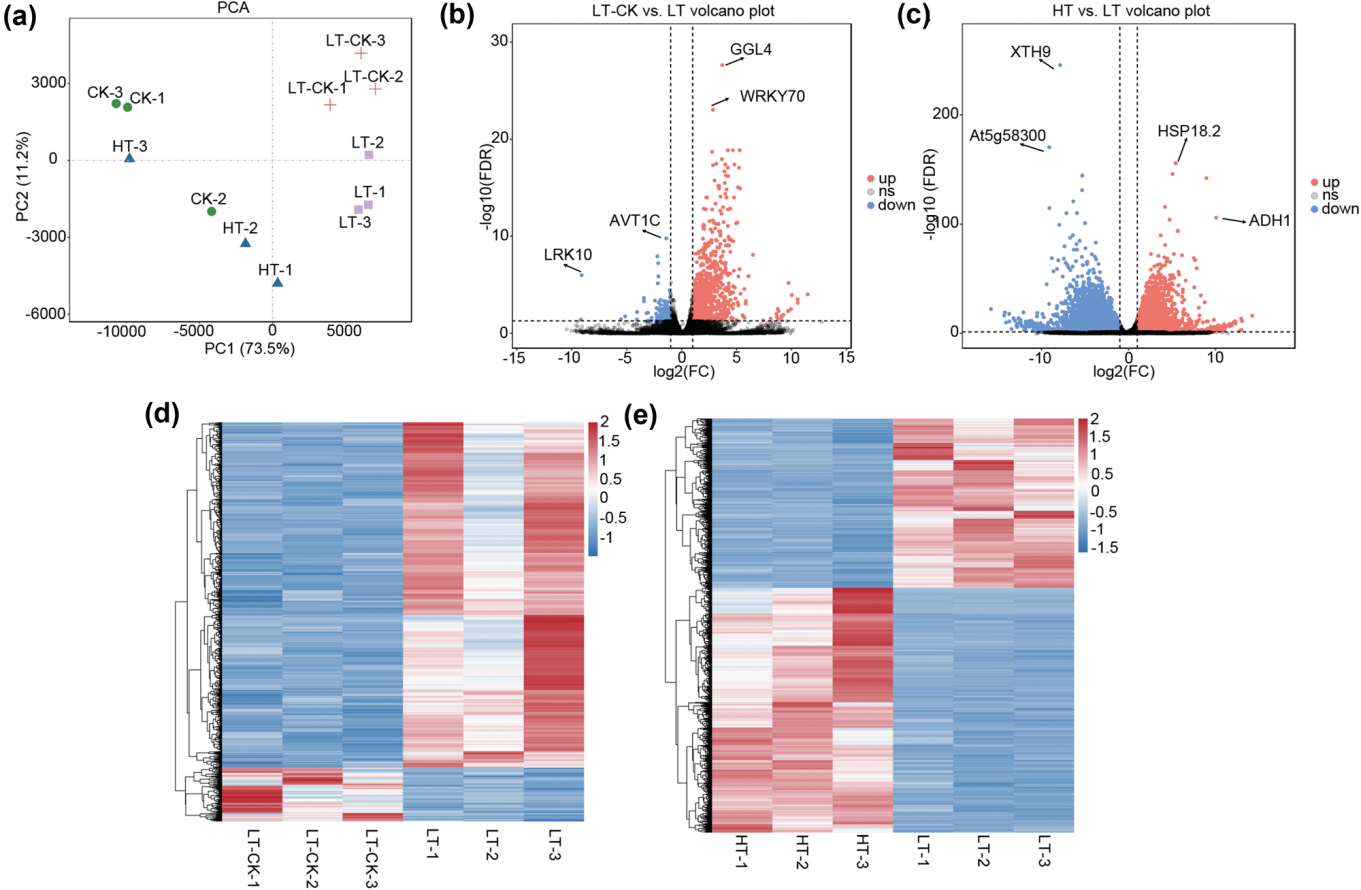
PCA and differential expression analysis. (a) PCA result for samples in three groups. (b) and (c) Volcano plots of DEGs in the two comparison groups. (d) and (e) Heatmaps of DEGs in the two comparison groups.

PCA revealed that the overall distribution characteristics of all samples in the four groups had a good separation status and less overlap with the samples in the other groups (Figure 2a). For differential expression analysis, we focused on DEGs in the LT-CK vs LT and HT vs LT groups. The former may explain the molecular mechanisms of NaHCO_3_ in the promotion of leaf senescence after picking, while the latter may explain the lack of significant effects of NaHCO_3_ on live tobacco leaves and the mechanisms of adaptation. Based on the LT-CK vs LT comparison, 1,134 DEGs (982 upregulated and 152 downregulated) were identified, while based on the HT vs LT comparison, 13,556 DEGs (5,190 upregulated and 8,366 downregulated) were identified. Volcano plots of the two groups of DEGs are shown in Figure 2b and c. Heatmaps of the DEGs are presented in Figure 2d and e.

### 3.4 Function analysis

**Figure 3:**
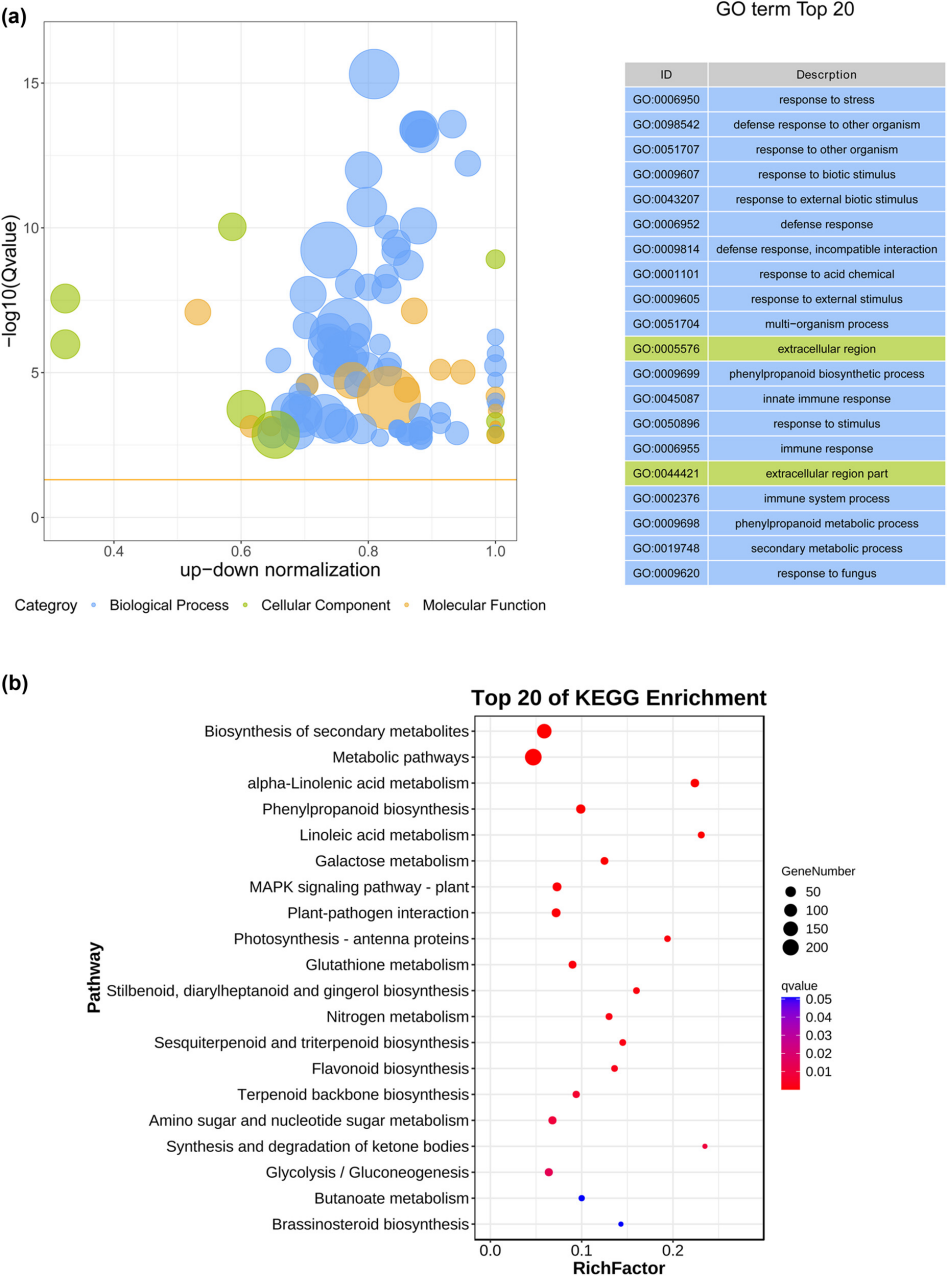
Function analysis of DEGs in LT-CK vs LT. (a) GO enrichment analysis and (b) KEGG enrichment analysis.

DEGs in the LT-CK vs LT groups were significantly associated with metabolic processes, cellular processes, response to stimuli, and biological regulation-related BP terms. In addition, these DEGs were enriched in MFs associated with catalytic activity, transporters, and activity, and CCs related to cells, organelles, and membranes (Figure 3a). Pathway enrichment analysis revealed that some biosynthesis-, metabolism-, and signaling-related pathways were enriched, such as alpha-linolenic acid metabolism, phenylpropanoid biosynthesis, linoleic acid metabolism, photosynthesis-antenna proteins, plant hormone signal transduction, and cysteine and methionine metabolism (Figure 3b).

**Figure 4:**
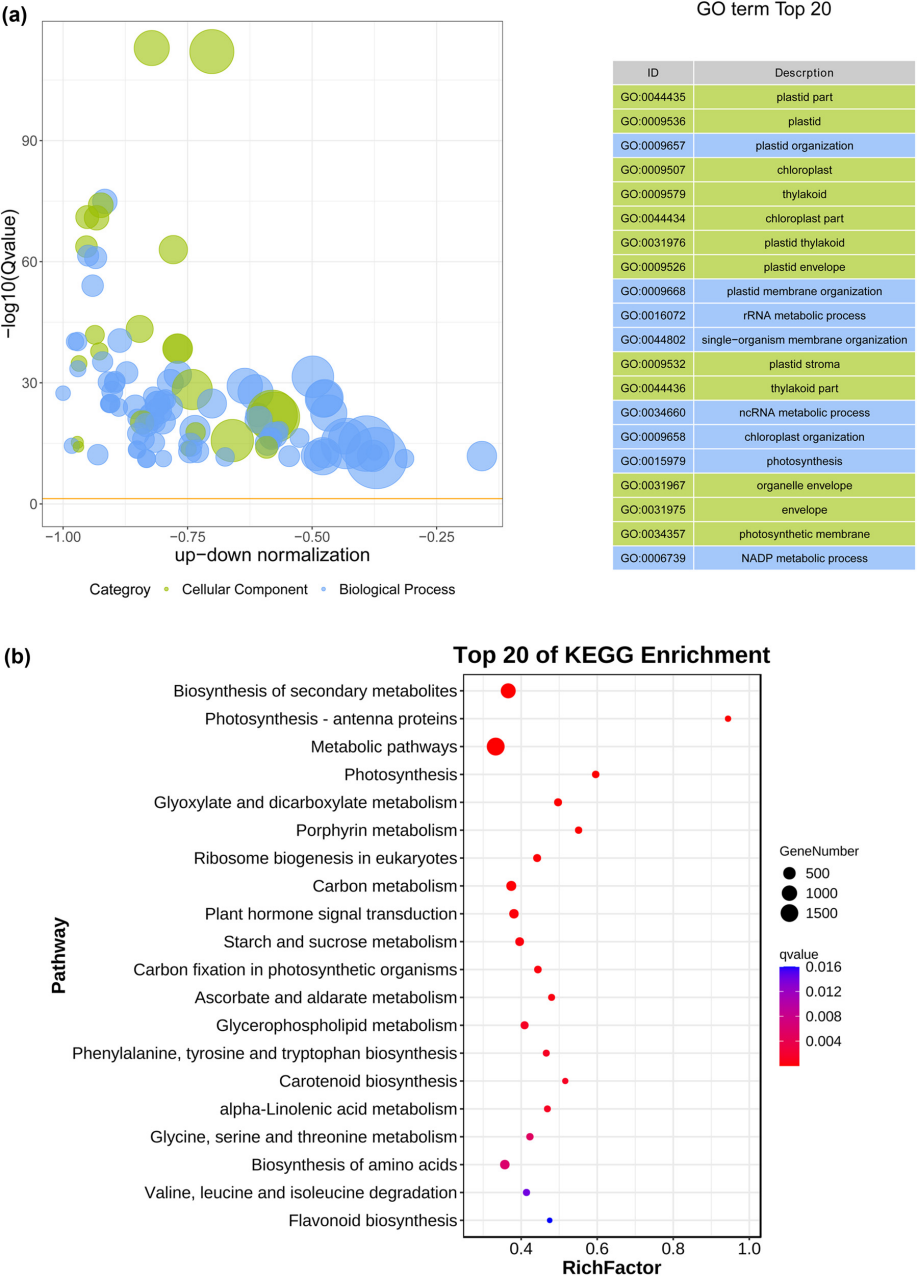
Function analysis of DEGs in HT vs LT. (a) and (b) GO enrichment analysis and (b) KEGG enrichment analysis.

The DEGs between HT and LT were enriched in BPs related to metabolic processes, cellular processes, and responses to stimuli; CCs related to organelles, cells, and membranes; and MFs related to catalytic activity, binding, and transporter activity (Figure 4a). These GO terms were similar to those of the LT-CK and LT groups. These genes were involved in pathways associated with photosynthesis-antenna proteins; metabolic pathways; photosynthesis; carbon metabolism; plant hormone signal transduction; phenylalanine, tyrosine, and tryptophan biosynthesis; glycine, serine, and threonine metabolism; and biosynthesis of amino acids (Figure 4b).

#### 3.4.1 GSEA

GSEA revealed that DEGs in LT-CK vs LT were positively correlated with GO terms for the negative regulation of peptidase activity, regulation of peptidase activity, defense response, incompatible interaction, and extracellular region and negatively correlated with photosystem, photosynthetic membrane, and thylakoid. The DEGs between HT and LT were negatively correlated with chloroplast organization, plastid membrane organization, photosynthesis, and pigment accumulation and positively correlated with seed maturation, dormancy, peroxisomal transport, developmental maturation, and mRNA catabolic processes. The ES GO terms with the top five highest and lowest NES are shown in Figure S1a–d.

Based on GSEA-KEGG, DEGs in LT-CK vs LT were positively correlated with pathways related to alpha-linolenic acid metabolism, proteasome, various types of *N*-glycan biosynthesis, glutathione metabolism, phenylalanine, tyrosine, and tryptophan biosynthesis and negatively correlated with photosynthesis-antenna proteins, photosynthesis, circadian rhythm-plant, phosphonate and phosphinate metabolism, and carotenoid biosynthesis. The DEGs in HT vs LT were positively correlated with autophagy-other, valine, leucine, and isoleucine degradation, MAPK signaling pathway, and endocytosis and negatively correlated with ribosome biogenesis in eukaryotes, photosynthesis-antenna proteins, DNA replication, proteasome, phenylalanine, tyrosine, and tryptophan biosynthesis. The ES pathways with the top five highest and lowest NES are shown in Figure S2a–d.

## 4 Discussion

In the present study, spraying NaHCO_3_ on tobacco leaves after harvesting significantly promoted leaf yellowing; this finding was completely evident after 3–4 days. However, the effect of spraying NaHCO_3_ on tobacco leaves before harvesting was not evident.

Chloroplast pigments are one of the main components affecting the quality and availability of tobacco leaves. These pigments also determine the color of tobacco leaves after modulation. The degradation products of chloroplast pigments are closely related to the aroma of tobacco leaves [20,21]. The change in color during tobacco leaf ripening is essentially a reflection of the change in the proportion of total chlorophyll and carotenoids to total chloroplast pigments in the leaf tissue. Salt stress and saline-alkaline stress usually reduce photosynthetic efficiency and inhibit chlorophyll synthesis in plants [22,23]. Consistent with these studies, we found that the tobacco leaves turned yellow after spraying with NaHCO_3._ During the long-term phylogenetic evolution of plants, protective mechanisms develop against the toxicity of ROS and free radicals in cells; the active oxygen scavenging system plays a major role among these mechanisms [24]. SOD, CAT, and POD are important protective enzymes in the active oxygen removal system that can remove excess ROS in plants, prevent the rapid accumulation of high concentrations of ROS, and prevent the peroxidation of membrane lipids [25]. MDA, the final product of lipid peroxidation, is widely used as an indicator of membrane damage caused by free radicals under abiotic stresses [26]. Physiological experiments revealed that in the detached leaves, the contents of Chl a, Chl b, and carotenoids and the activities of CAT and SOD decreased significantly, while the activity of POD increased after spraying with NaHCO_3_. The MDA content also increased after spraying with NaHCO_3_. However, the effect of spraying NaHCO_3_ on tobacco leaves before harvesting was not evident. According to Wang et al., NaHCO_3_ can induce a ROS burst, which damages the growth and photosystems of tobacco [27]. In the livers of crucian carp, the SOD, POD, and CAT activities and MDA content were significantly increased by 40 and 60 mmol/L NaHCO_3_ [28]. We speculate that the decrease in SOD and CAT was due to the inability of detached leaves to produce SOD for ROS clearance. Our study further highlights the effects of NaHCO_3_ on the promotion of tobacco leaf maturation and senescence after harvesting.

We proceeded to determine the potential molecular mechanisms whereby NaHCO_3_ promotes the maturity of tobacco leaves using RNA-seq. Upon comparing the LT-CK and LT groups, 1,134 DEGs were identified. Pathway enrichment analysis revealed that several biosynthesis-, metabolism-, and signaling-related pathways were also enriched. For instance, alpha-linolenic acid metabolism, various types of *N*-glycan biosynthesis, cysteine and methionine metabolism, and phenylalanine, tyrosine, and tryptophan biosynthesis were upregulated, whereas photosynthesis and carotenoid biosynthesis were downregulated after NaHCO_3_ treatment. Methionine is a precursor of *S*-adenosyl methionine, which is a precursor of ethylene [29]. The upregulation of cysteine and metabolism of methionine may promote ethylene production and accelerate the maturity and senescence of tobacco leaves. Changes in amino acid metabolism, especially the increased synthesis of aspartic acid and asparagine, are related to maturation, senescence, ammonia accumulation, and detoxification in plants [30]. Alpha-linolenic acid metabolism produces the precursors for jasmonic acid biosynthesis [31]. Jasmonic acid promotes leaf senescence in *Arabidopsis* [32]. Interrupting jasmonic acid signaling or inhibiting its biosynthesis delays plant leaf senescence [33]. Altogether, NaHCO_3_ may promote the maturity and senescence of tobacco leaves by upregulating amino acid and alpha-linolenic acid metabolism and downregulating photosynthesis and carotenoid biosynthesis.

A total of 13,556 DEGs were identified in the HT vs LT groups. These DEGs were positively correlated with autophagy-other, valine, leucine, and isoleucine degradation, ether lipid metabolism, and MAPK signaling pathways and negatively correlated with photosynthesis-antenna proteins, DNA replication, proteasomes, phenylalanine, tyrosine, and tryptophan biosynthesis. During leaf senescence, autophagic activity increases to remove damaged organelles, oxidized proteins, and other toxic compounds from cells [27]. The upregulation of the autophagy pathway may explain the inability to identify the effect of NaHCO_3_ on live tobacco leaves. Interestingly, among these pathways, phenylalanine, tyrosine, and tryptophan biosynthesis had opposite effects in the two comparison groups. Phenylalanine, tyrosine, and tryptophan are aromatic amino acids [34]. A previous study revealed that aromatic amino acids accumulate during leaf senescence [35]. Mou et al. [36] also suggested that amino acids and derivatives, such as tryptophan, proline, tyrosine, and phenylalanine, accumulate significantly during leaf senescence. The accumulation of these amino acids is a strategy for maintaining osmotic balance during leaf senescence. In addition to aromatic amino acids, the contents of valine, leucine, and isoleucine significantly increase during late senescence [37]. Therefore, upregulation of the valine, leucine, and isoleucine degradation pathways may also be one of the adaptive mechanisms of live tobacco leaves after treatment with NaHCO_3_. The MAPK signaling pathway is an important signal transduction pathway in plants and has been suggested to be associated with *Arabidopsis* leaf senescence [38]. Overall, we speculate that live tobacco leaves may adapt to NaHCO_3_ stress via amino acid degradation, increasing autophagic activity and regulating some signaling pathways.

In the production process, tobacco plants might mature late due to abnormal climate or improper fertilizer operation. Spraying NaHCO_3_ on the leaves after harvesting can induce the aging and yellow effect of detached leaves of flue-cured tobacco. The main reason is that exogenous NaHCO_3_ promotes the metabolism of macromolecular substances in flue-cured tobacco and increases the coordination of substances contained in tobacco leaves. The curing properties of refractory yellow tobacco were improved, and the chemical composition and aroma quality of the cured tobacco were positively affected. Exogenous NaHCO_3_-induced leaf aging greatly reduced the influence of climate on flue-cured tobacco quality. The effect of exogenous NaHCO_3_ on the maturation and senescence of field flue-cured tobacco, the application of detached leaf senescence technology in field production, and the effect of spraying NaHCO_3_ on the quality of cured tobacco leaves still need further research.

## 5 Conclusion

To our knowledge, this is the first study to elucidate the molecular and metabolic mechanisms by which NaHCO_3_ promotes the maturation of tobacco leaves. NaHCO_3_ may enhance amino acid and alpha-linolenic acid metabolism and weaken photosynthesis to promote the maturity and senescence of tobacco leaves after harvesting. Therefore, NaHCO_3_ might be a good exogenous substance that promotes the maturation of tobacco leaves and the conversion of nicotine to nornicotine after harvesting. Live tobacco leaves may adapt to NaHCO_3_ stress through amino acid degradation, increasing autophagic activity and regulating some signaling pathways. However, further experiments are required to confirm our results.

## Supplementary Material

Supplementary Figure
