# Supplementary material for: Transcriptome analysis reveals the mechanism of NaHCO3 promoting tobacco leaf maturation
Source: Open Life Sci. 2024 Apr 15;19(1):20220849. doi: 10.1515/biol-2022-0849 (PMC11022121; doi:10.1515/biol-2022-0849)
Supplement: Supplementary Figure [file biol-2022-0849-sm.pdf]

Supplementary material

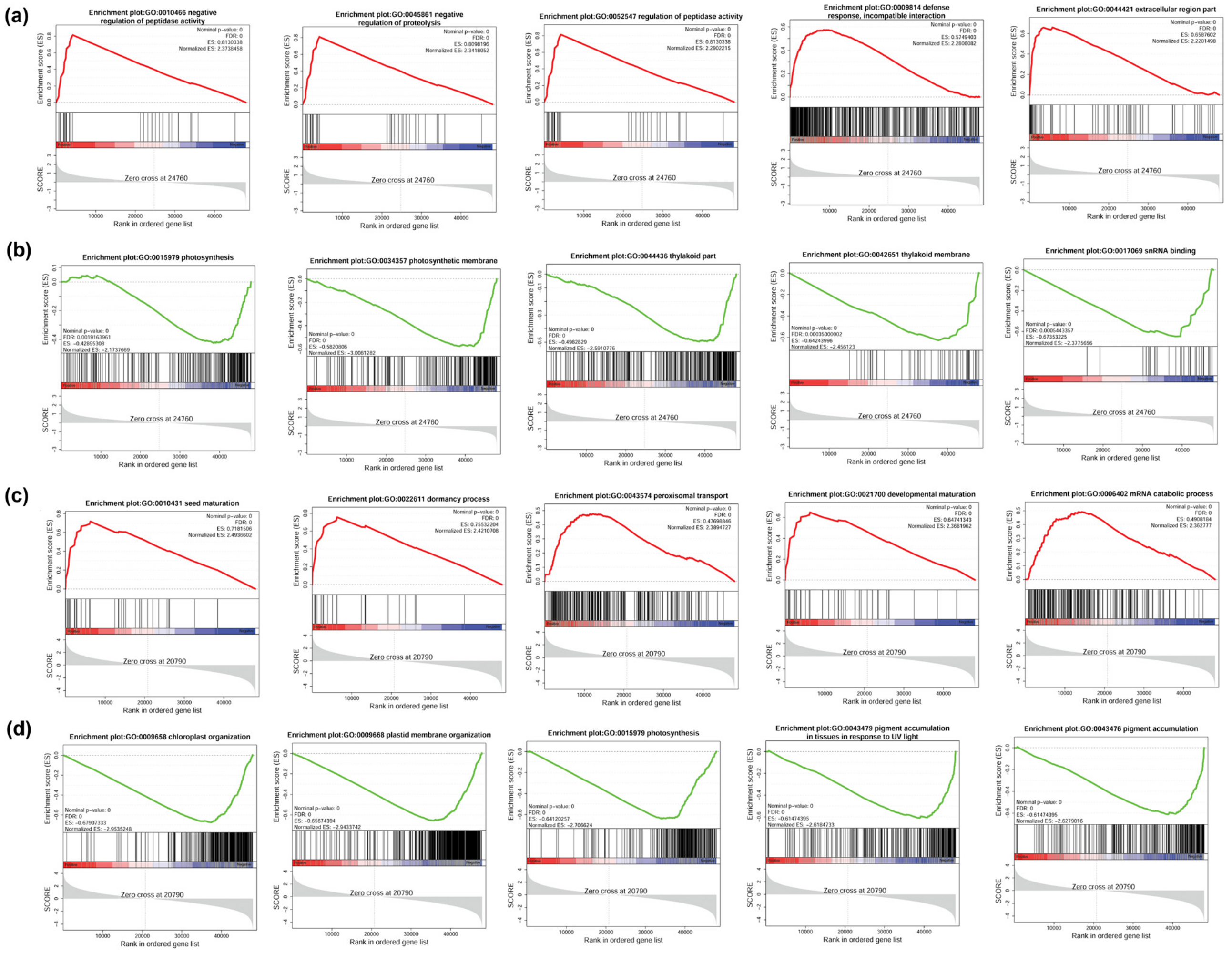

Figure S1: Results of GSEA based on GO. (a) and (b) Top 5 positively and negatively correlated GO terms for DEGs in LT-CK vs LT. (c) and (d) Top 5 positively and negatively correlated GO terms for DEGs in HT vs LT.

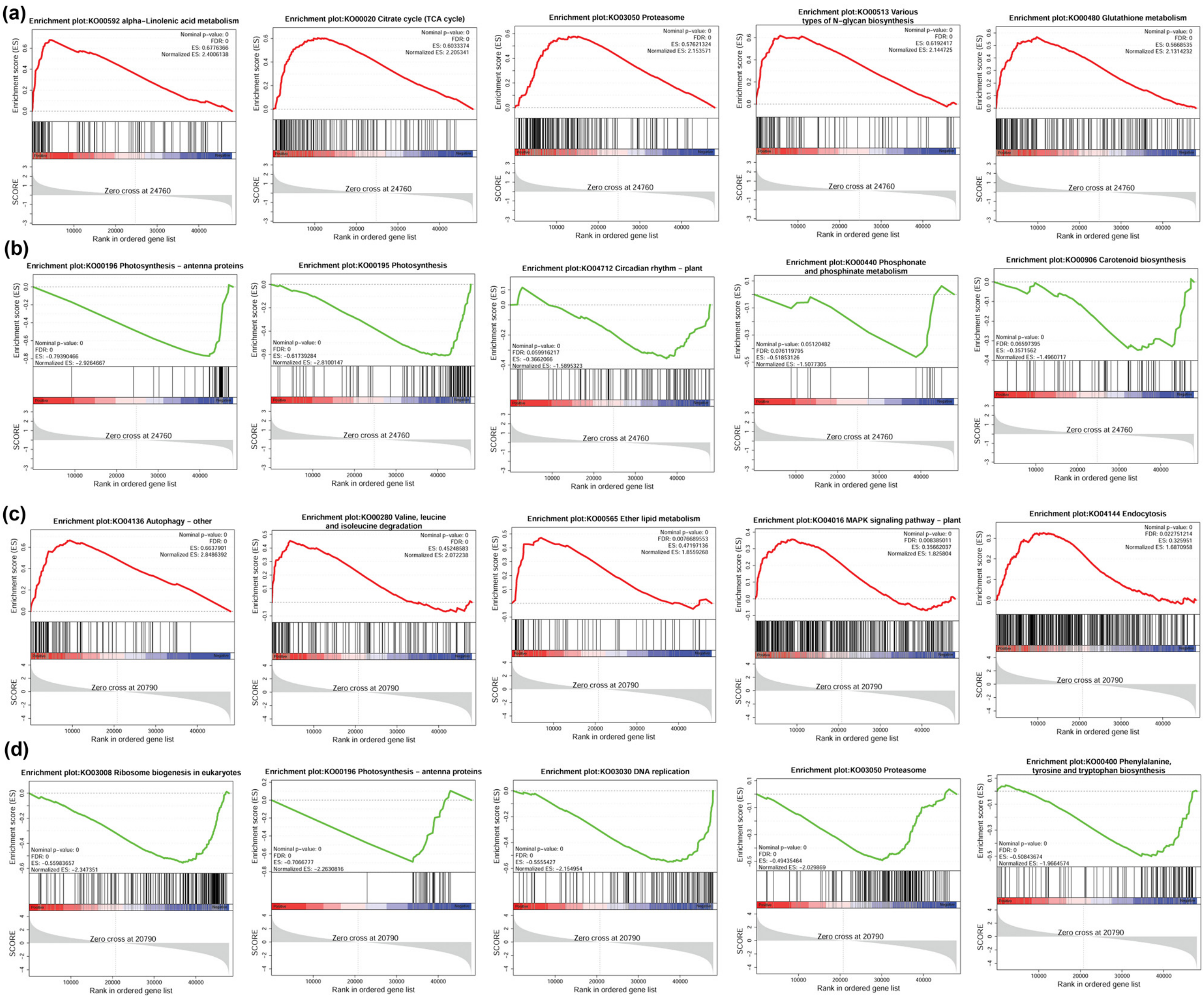

Figure S2: Results of GSEA based on KEGG. (a) and (b) Top 5 positively and negatively correlated pathways for DEGs in LT-CK vs LT. (c) and (d) Top 5 positively and negatively correlated pathways for DEGs in HT vs LT.
